# Music Perception Abilities and Ambiguous Word Learning: Is There Cross-Domain Transfer in Nonmusicians?

**DOI:** 10.3389/fpsyg.2022.801263

**Published:** 2022-02-28

**Authors:** Eline A. Smit, Andrew J. Milne, Paola Escudero

**Affiliations:** ^1^The MARCS Institute for Brain, Behaviour and Development, Western Sydney University, Sydney, NSW, Australia; ^2^ARC Centre of Excellence for the Dynamics of Language, Canberra, ACT, Australia

**Keywords:** music perception, pitch, phonological processing, cross-situational word learning, auditory perception

## Abstract

Perception of music and speech is based on similar auditory skills, and it is often suggested that those with enhanced music perception skills may perceive and learn novel words more easily. The current study tested whether music perception abilities are associated with novel word learning in an ambiguous learning scenario. Using a cross-situational word learning (CSWL) task, nonmusician adults were exposed to word-object pairings between eight novel words and visual referents. Novel words were either non-minimal pairs differing in all sounds or minimal pairs differing in their initial consonant or vowel. In order to be successful in this task, learners need to be able to correctly encode the phonological details of the novel words and have sufficient auditory working memory to remember the correct word-object pairings. Using the Mistuning Perception Test (MPT) and the Melodic Discrimination Test (MDT), we measured learners’ pitch perception and auditory working memory. We predicted that those with higher MPT and MDT values would perform better in the CSWL task and in particular for novel words with high phonological overlap (i.e., minimal pairs). We found that higher musical perception skills led to higher accuracy for non-minimal pairs and minimal pairs differing in their initial consonant. Interestingly, this was not the case for vowel minimal pairs. We discuss the results in relation to theories of second language word learning such as the Second Language Perception model (L2LP).

## Introduction

Music and language are universal to humans (Patel, 2003) and the connection between the two has been an object of research for centuries, with early ideas even suggesting that music is a spin-off of language in evolution (Pinker, 1997). While the precise origins of music and language remain unclear, there are many parallels that can be drawn between the two. Both use a rule-based hierarchical structure organized into discrete elements and sequences (Tervaniemi et al., 1999; Tervaniemi, 2001; Patel, 2003; Degé and Schwarzer, 2011; Burnham et al., 2015), such as syllables, words, and sentences for language and single notes, intervals, chords, and musical phrases for music (Ong et al., 2016). When focusing on the acoustic characteristics of music and speech sounds, similarities can be found in the reliance on segments of rhythm and harmony alternated with silence, pitch, acoustic envelope, duration, and fundamental frequency (Varnet et al., 2015). In order to understand music and speech, a listener needs to categorize sounds into meaningful units. For speech, perceptual skills are needed to distinguish sounds into separate vowels or consonants and for music into pitches (Hallam, 2017). The auditory skills needed to process language are similar to those needed to discriminate between rhythms (Lamb and Gregory, 1993), harmonies, and melodies (Barwick et al., 1989; Lamb and Gregory, 1993; Anvari et al., 2002). Numerous studies support the overlap of auditory processes involved in music and speech perception (Overy, 2003; Tallal and Gaab, 2006; Patel and Iversen, 2007; Sammler et al., 2007; Wong and Perrachione, 2007; Chandrasekaran et al., 2009; Kraus and Chandrasekaran, 2010; Besson et al., 2011; Rogalsky et al., 2011; Schulze et al., 2011; Bidelman et al., 2013; Gordon et al., 2015; Kraus and White-Schwoch, 2017) and individuals with musical training appear to be advantaged in these shared processes (Krishnan et al., 2005; Bigand and Poulin-Charronnat, 2006; Krizman et al., 2012; White-Schwoch et al., 2013; Elmer et al., 2014).

Those that are expert listeners in either music or language have been found to show *cross-domain transfer* (Ong et al., 2016), where an advantage is found for perception in the other domain; for example, in word segmentation (François et al., 2013), syllabic perception (Musacchia et al., 2007; Ott et al., 2011; Elmer et al., 2012; Kühnis et al., 2013; Chobert et al., 2014; Bidelman and Alain, 2015), receptive and productive phonological skills at the word, sentence and passage level (Slevc and Miyake, 2006), and word dictation (Talamini et al., 2018). It is suggested that long-term expertise in music, which is gained by years of practice, has led to a fine-tuning of the auditory system (Strait and Kraus, 2011a,b), as evidenced by enhanced neural responses to changes in acoustic elements, such as pitch, intensity, and voice onset time (Schön et al., 2004; Magne et al., 2006; Jentschke and Koelsch, 2009; Marie et al., 2011a,b). Musicians indeed show enhanced cortical processing of pitch in speech compared to nonmusicians (Magne et al., 2006; Besson et al., 2007; Musacchia et al., 2007; Kraus and Chandrasekaran, 2010). These and numerous other studies support the idea of cross-domain transfer between music and speech perception (see Hallam, 2017 for an extensive list). The present study focuses on the potential auditory processing advantages in pitch perception and auditory working memory (Ott et al., 2011; Kühnis et al., 2013; Pinheiro et al., 2015; Dittinger et al., 2016, 2017, 2019) associated with music perception skills. Many examples of the effect of music training on speech processing have been reported. For instance, training in music has been associated with phonological perception in the native language (L1; Zuk et al., 2013) and with fluency in a second language (L2; Swaminathan and Gopinath, 2013; Yang et al., 2014). As well, longitudinal studies in children’s speech perception found positive effects of music training (Moreno et al., 2009; Degé and Schwarzer, 2011; François et al., 2013; Thomson et al., 2013). Regarding the transfer of music experience to word learning, Dittinger et al. (2016, 2017, 2019) presented listeners with unfamiliar Thai monosyllabic words and familiar visual referents during a learning phase and tested them on their ability to match the words with their corresponding visual objects. Overall, they found that both music training led to higher accuracy in both young adults and children. Additionally, a longitudinal effect of music training was shown, as musicians had the same advantage when tested 5 months later (Dittinger et al., 2016).

However, counter-examples to a positive association between music training and speech perception also exist (Ruggles et al., 2014; Boebinger et al., 2015; Swaminathan and Schellenberg, 2017; Stewart and Pittman, 2021). For instance, Swaminathan and Schellenberg (2017) found that rhythm perception skills predicted English listeners’ discrimination of Zulu phonemic contrasts, but only for contrasts that closely resembled English phonemic contrasts. The authors found no association between other music perception skills, such as melody perception or general music training and non-native speech perception, suggesting that an effect of rhythm rather than pitch is related to participants’ native language background rather than their music skills. Specifically, unlike for tonal languages, English does not contrast pitch for signaling lexical meaning; hence, it is likely that listeners focus on other cues, such as temporal cues, to distinguish one word from another.

Apart from the ability to perceive novel or familiar phonological contrasts, another important component involved in speech processing, including novel word learning, is working memory. Working memory, which is a short-term memory involved in immediate conscious perceptual and linguistic processing, plays an important role in novel word learning (Gathercole et al., 1997; Warmington et al., 2019). Mixed results have been found regarding a musician’s advantage in working memory, with some studies finding no difference between musicians and nonmusicians (Hansen et al., 2012), whereas others find improved auditory and verbal working memory for musicians compared to nonmusicians (Parbery-Clark et al., 2011; Bergman Nutley et al., 2014). A meta-analysis conducted by Talamini et al. (2017) on different types of memory found a medium effect size for short-term and working memory with musicians performing better than nonmusicians, depending on the type of stimulus used.

Most studies examining the link between speech processing and musical abilities have compared professional musicians to nonmusicians (see Zhu et al., 2021), with a large focus on explicit tasks when comparing linguistic and musical abilities (e.g., Dittinger et al., 2016, 2017, 2019). In such tasks, there is no ambiguity during learning, but the link between words and meaning in daily life is much more ambiguous without immediate clear connections, with studies showing that pairing between words and their referent objects are learned by tracking co-occurrences through repeated exposure (e.g., Smith and Yu, 2008; Escudero et al., 2016b; Mulak et al., 2019). Very little is known about the role of musical abilities for ambiguous word learning scenarios, which are most common in everyday life of word learning (Tuninetti et al., 2020). In the realm of music perception, recent studies have shown that musical elements, such as musical grammar (Loui et al., 2010), harmony (Jonaitis and Saffran, 2009), musical expectation (Pearce et al., 2010), and novel pitch distributions from unfamiliar musical scales (Ong et al., 2017a; Leung and Dean, 2018), can be learned through statistical learning. Statistical learning is a domain-general learning mechanism leading to the acquisition of statistical regularities in (in this case auditory) input. This type of learning may lead to cross-domain transfer between music and language due to learners showing sensitivity toward particular acoustic cues (e.g., pitch; Ong et al., 2016) which may result in improved ambiguous word learning. Despite the potential effect of music abilities on ambiguous word learning and the many types of learners considered in statistical word learning studies (such as young infants, children and adults, and L2 learners Yu and Smith, 2007; Smith and Yu, 2008; Suanda et al., 2014; Escudero et al., 2016b,c; Mulak et al., 2019), participants’ musical experience or expertise have yet to investigated. In sum, it has been established that music and language rely on similar general auditory processing skills and, although results are mixed, the majority of studies finds an advantage for music training on auditory and speech perception. By testing whether music abilities in a nonmusician population can help ambiguous word learning, we can further unravel more influences of music on language learning than previously shown.

The current study tests the effect of specific music perception abilities on statistical learning of novel words in a nonmusician adult population. We tested musical abilities through two adaptive psychometric tests targeting specific music perception skills, namely, the ability to perceive fine-pitch mistuning, through the Mistuning Perception Test (MPT; Larrouy-Maestri et al., 2018, 2019), and the ability to discriminate between pitch sequences, through the Melodic Discrimination Test (MDT; Harrison et al., 2017; Harrison and Müllensiefen, 2018). The MPT is an adaptive psychometric test measuring sensitivity to intonation accuracy in vocal musical performance (Larrouy-Maestri et al., 2018, 2019). Perception of vocal mistuning is a core musical ability, as evidenced by its high correlation with other musical traits (Law and Zentner, 2012; Kunert et al., 2016; Larrouy-Maestri et al., 2019), and its importance when judging the quality of a musical performance (Larrouy-Maestri et al., 2019). The MDT aims to test melodic working memory, as it requires melodies to be held in auditory working memory in order for participants to compare and discriminate them correctly (Dowling, 1978; Harrison et al., 2017; Harrison and Müllensiefen, 2018). To do well in these tasks, specific auditory processing skills, in particular pitch perception and auditory working memory, are required. A recent large-scale study across thousands of speakers of tonal, pitch-accented, and non-tonal languages using these two tasks (and a beat alignment task) has shown that language experience shapes music perception ability (Liu et al., 2021). Here, we test the opposite, namely, whether the same music perception skills help with language learning, and specifically when learning novel words with different degrees of phonological overlap. Our specific focus is on pitch processing abilities but acknowledge that rhythm processing is also an important component in music and language processing (see Swaminathan and Schellenberg, 2017).

To test whether pitch perception and auditory working memory are helpful when learning words in ambiguous scenarios, we used a cross-situational word learning (CSWL) paradigm in which meanings of new words are learned through multiple exposures over time without explicit instruction, where learning of word-object pairings can only take place through their statistical co-occurrences (e.g., Escudero et al., under review; Yu and Smith, 2007; Kachergis et al., 2010; Smith and Smith, 2012; Escudero et al., 2016a,b, 2021; Mulak et al., 2019; Tuninetti et al., 2020). Early CSWL experiments focused on words with very little phonological overlap (e.g., Smith and Yu, 2008; Vlach and Johnson, 2013), where a listener can rely on other cues to learn the novel words and does not have to focus on the fine phonological details of each word (Escudero et al., 2016b). Therefore, (Escudero et al., 2016a,b) and Mulak et al. (2019) studied CSWL of monosyllabic non-minimal and minimal pairs, differing only in one vowel or consonant, to test whether listeners can encode sufficient phonological detail in a short time to learn these difficult phonological contrasts. It was found that accurate phonological encoding of vowel and consonant contrasts predicts high performance in CSWL tasks (Escudero et al., 2016a; Mulak et al., 2019).

In the present study, we thus tested whether musical ability impacts word learning of phonologically overlapping words using Escudero et al. (2016b) and Mulak et al. (2019)’s CSWL paradigm. Overall, we hypothesize that those with stronger musical abilities are better at perceiving speech sounds due to enhanced pitch perception and working memory, and that will be reflected in higher accuracy overall in the CSWL task. We may also see differences in how well vowels and consonants are learned, due to higher acoustic variability in vowels compared to consonants (Ong et al., 2015), which may favor learners with stronger pitch perception skills.

## Materials and Methods

### Participants

Fifty-four participants took part in the study and were tested online, which is our common practice since the start of the COVID-19 pandemic, using our validated online testing protocols (Escudero et al., 2021). In Escudero et al. (2021), we compared online and face-to-face testing using the same CSWL design and online testing results were found to be very similar to results from the laboratory. Ten participants were excluded from the analysis due to technical difficulties, mostly internet dropouts during the experiment or excessive environmental noise, leading to a total participant sample of 44 (*M*_age_ = 26.79, *SD*_age_ = 11.12, 33 females). Participants were recruited through the Western Sydney University’s online research participation system (SONA) or *via* word-of-mouth and participation was rewarded with course credit for the former and voluntary for the latter. Written informed consent was obtained online from all participants prior to the start of the experiment, and the study was approved by the Western Sydney University Human Research Ethics Committee (H11022).

### Materials

#### Questionnaires

The questionnaires conducted at the beginning of the experiment consisted of two parts: a language and a musical background questionnaire. The language background questionnaire consisted of questions aimed to get detailed information regarding participants native (and other) language, as well as the language background of their parents/caretakers. The musical background questionnaire is the Goldsmiths Musical Sophistication Index (GMSI; Müllensiefen et al., 2014), which aims to collect wide-range data related to one’s engagement with music (e.g., music listening and music performance behavior). Both questionnaires were administered through Qualtrics (Qualtrics, Provo, UT). From the GMSI, 23 participants indicated having zero years of experience with playing an instrument, and seven had 10 or more years of experience. From the language questionnaire, we found that 17 were Australian English monolinguals and 27 were bi- or multilinguals.

#### Cross-Situational Word Learning

All words and visual referents have been used in prior CSWL studies (Vlach and Sandhofer, 2014; Escudero et al., 2016a,b; Mulak et al., 2019; Escudero et al., under review). Novel words consisted of eight monosyllabic nonsense words recorded by a female native speaker of Australian English and followed a consonant-vowel-consonant (CVC) structure while adhering to English phonotactics. The stimuli were produced in *infant-directed speech* (IDS) as we are replicating previous studies that used IDS to compare adult and infant listeners and included two tokens for each word to match prosodic contours across all stimuli (Escudero et al., 2016a,b).

The eight words were combined into minimal pair sets to form specific consonant or vowel minimal pairs or non-minimal pairs. The two types of minimal pairs featured words that either differed in their initial consonant (consMPs; e.g., BON-TON) or in their vowel (vowelMPs; e.g., DIT-DUT). Non-minimal pairs were formed by pairing two words from each of the two minimal pair types in random order (nonMPs; e.g., BON-DIT).

**Figure 1:**
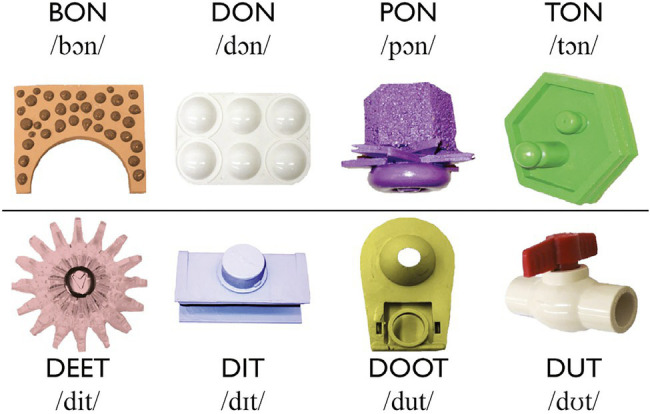
The eight novel words and their visual referents. The four words in the top row are minimally different in their initial consonant, whereas the words on the bottom are minimally different in their vowel. The vowel used for the consonant minimal pairs is/O/as in POT. Vowels used for the vowel minimal pairs are/i/as in BEAT, /I/as in BIT, /u/as in BOOT, and/U/as in PUT.

Every novel word was randomly paired with a color picture of a novel item, which is not readily identifiable as a real-world object. These word-referent pairings were the same for all participants. An overview of the novel words and visual referents is presented in Figure 1.

#### Mistuning Perception Test

The MPT, which is an adaptive psychometric test, uses short excerpts (6–12 s) of musical stimuli from pop music performances which are representative of real-life music and are therefore ecologically valid (from MedleyDB; Bittner et al., 2014). The test highly correlates with low- and high-level pitch perception abilities, such as pitch discrimination and melody discrimination, and thus provides an assessment of important pitch processing abilities (Larrouy-Maestri et al., 2019). In a two-alternative forced-choice task, participants were presented with a pitch-shifted version (out-of-tune) and the normal version (in-tune) of a stimulus and were asked to indicate which version was out-of-tune. Pitch shifting varied from 10 cents to 100 cents, sharp, and flat (for more details about the construction of the MPT, see Larrouy-Maestri et al., 2019). Before starting the task, participants received an example of an out-of-tune and an in-tune version. A demo of the experiment can be found on https://shiny.gold-msi.org/longgold_demo/?test=MPT.

#### Melodic Discrimination Test

Similar to the MPT, the MDT is also an adaptive psychometric test. The MDT is developed to test one’s ability to discriminate between two melodies (Harrison et al., 2017; Harrison and Müllensiefen, 2018). Participants are presented with a three-alternative forced-choice (3-AFC) paradigm where they listen to three different versions of the same melody, each with a different pitch height (musical transposition), and with one containing an *altered* note produced by changing its relative pitch compared to the base melody (Harrison et al., 2017), resulting in a pitch height change for one note compared to the other melodies. Each melody can be altered using four pre-determined constraints: (1) melodies with five notes or fewer cannot have the first nor last note altered, (2) melodies with six notes or longer cannot have the first two nor last two notes altered, (3) the note cannot be altered by more than six semitones, and (4) the altered not must be between an eight note and a dotted half note in length (see Harrison et al., 2017). Participants are asked to indicate which of the three melodies are the odd one out. Participants heard an implementation of the MDT with 20 items (see doi:10.5281/zenodo.1300951) using the shiny package in R (Chang et al., 2020) which uses an adaptive item selection procedure with each participant’s performance level determining the level of difficulty of item presentation. Performance level is estimated using Item Response Theory (de Ayala, 2009). A demo of the experiment can be found on https://shiny.gold-msi.org/longgold_demo/?test=MDT. Tests scores for both the MDT as the MPT are computed as intermediate and final abilities with weighted-likelihood estimation (Warm, 1989) and using Urry’s rule for item selection (Magis and Raîche, 2012).

### Procedure

We followed our adult online testing protocol, which was validated in Escudero et al. (2021), for details please see on https://osf.io/nwr5d/. In short, participants signed up for a timeslot on SONA after which they received an email with specific instructions for the experiment (e.g., wearing headphones and participating from a silent study space with no background noise was required) and an invitation for a Zoom call. Participants unable to meet the participation requirements were excluded from the analysis (see Section “Participants”). During the Zoom call, participants were first familiarized with the procedure and then sent links to the consent forms, background questionnaires, and the experiment. During the experiment, they were asked to share their screen and computer audio throughout the entire video call, apart from when filling out the questionnaire to ensure privacy. Participants’ screen and audio sharing enabled experimenter’s verification of appropriate auditory stimuli presentation and participants’ attention. The experimenter was on mute and with their video off during the experiment to avoid experimenter bias.

Participants first completed the language and musical background questionnaires and were then instructed to start the CSWL task. The CSWL task consisted of a learning and a test phase set up in PsychoPy 3 (Peirce, 2007; Peirce et al., 2019) hosted on Pavlovia.org. Following previous CSWL studies, minimal instruction was provided (i.e., “Please listen to the sounds and look at the images”) prior to the learning phase. During the learning phase, participants saw 24 trials each consisting of two images accompanied by auditory representations of two words without indication of which word corresponded to which image. The visual referents were presented first for 0.5 s before the onset of the first word. Both words lasted for 1 s and were followed by a 0.5 s inter-stimuli interval (ISI). After this, a 2 s inter-trial interval (IT) consisting of a blank screen was then presented, leading to a total trial time of 5 s. The learning phase was directly followed by a test phase of 24 trials, for which participants were told that they would be tested on what they have learned and to indicate their answers by pressing specific keys on the keyboard. Every test trial presented two possible visual referents simultaneously on the screen for 3 s. During this, participants heard one spoken target word four times (with alternating tokens of the words) and were then asked to indicate which visual referent (the left or the right one) corresponded with the target word by pressing a key on the keyboard any time after the onset of the target word. Trial order was randomized across all participants. The presentation of left and right of the visual referents was counterbalanced and resulted in two between-subject learning conditions. A blank screen of 2 s was presented in between trials. Directly after the CSWL task, participants completed the MDT and the MPT task to measure their music perception abilities.

## Statistical Analysis

We used a Bayesian Item Response Theory (IRT) model to analyze accuracy. IRT models are particularly useful for predicting the probability of an accurate answer depending on an item’s difficulty, its discriminability, a participant’s latent ability, and a specified guessing parameter (Bürkner, 2020), which provides a lower bound for the model’s predictions. The statistical analyses were run in the statistical program R (R Core Team, 2020) with the brms package using Stan (Bürkner, 2017, 2018; R Core Team, 2020).

We used approximate leave-one-out (LOO) cross-validation to find the model that generalizes best to out-of-sample data. Additionally including GMSI or participant’s language background did not improve the out-of-sample predictions of the model.

The best model included only the interaction between Pair type and MPT. However, as we are interested in both MPT and MDT as main factors, we will report the next best model. The difference in the LOOIC values for these two models is negligible. Prior to fitting the models, we tested for correlation between MPT, MDT, and GMSI. MPT and MDT were moderately positively correlated, *r*(1054) = 0.39, *p* < 0.005; MPT and GMSI were moderately positively correlated, *r*(1054) = 0.30; and MDT and GMSI were weakly positively correlated, *r*(1054) = 0.11.

Accuracy was modeled as a binary response variable, with 0 for inaccurate and 1 for accurate. We used a 4-parameter non-linear logistic model (4PL, Agresti, 2010) on the Bernoulli distribution with an item, a person and a guessing parameter. The discriminability parameter is removed. The item parameter models the difficulty of the tested items (in this case the pair types); the person parameter models the individual ability of each participant. The guessing parameter represents the probability of being accurate if participants were only guessing (Bürkner, 2020). All of our trials are binary forced choice; hence, we use a fixed guessing parameter of 0.5. An advantage of using IRT for modeling binary accuracy responses is that this probability can be taken into account as a type of baseline in the model, meaning that the model’s estimates of the underlying probability of being correct will not fall below the 0.5 threshold. We did not include a discrimination parameter, as all tested items are very similar.

The categorical variable Pair type was turned into a factor and modeled using dummy coding, which is the default in R. For MPT and MDT, we are using the raw data scores, as recommended by the experiment designers (MPT: Larrouy-Maestri et al., 2018, 2019; MDT: Harrison et al., 2017; Harrison and Müllensiefen, 2018), which were computed from the underlying item response models. These scores range from −4 to +4. GMSI was scaled and centered to a previously determined population mean from Harrison and Müllensiefen (2018).

For the 3-PL IRT accuracy model, we included separate priors for the item, person and guessing parameters. As detailed below, all such priors were weakly informative in that they weakly favor an effect of zero size and disfavor unfeasibly large effects. The following model formula (including priors) was run in R:


Accuracy  ~0.5  +  0.5  ^*^  inv_logit(eta),
Eta  ~1  +  Pair type  ^*^  (MDT  ability  +  MPT ability) + (1|item) + (1|participant),
nl = TRUE)
family  <−  brmsfamily(“bernoulli,” link  =  “identitiy”).
priors<−
prior(“normal  (0,5),”  class  =  “b,”  nlpar   =  “eta”)  +
prior(“constant(1),”  class  =  “sd,” group  =  “participant,” nlpar  =  “eta”)  +
prior(“normal(0,3),”  class  =  “sd,”  group  =  “item,” nlpar  =  “eta”).


An important aspect of Bayesian regression is that it calculates the whole posterior distribution of each effect, which allows for the calculation of credibility intervals. In contrast with frequentist confidence intervals, credibility intervals indicate the 95% certainty that reported effect falls within the range of the interval (Smit et al., 2019). Evidence for a hypothesized effect will be assessed through evidence ratios, which quantify the likelihood of a tested hypothesis against its alternative (Bürkner, 2017, 2018). We consider evidence ratios of >10 to be strong evidence and above >30 to be very strong evidence [see Jeffreys (1998), as cited by Kruschke (2018)]. For directional hypotheses, where the predicted direction of an effect is given, effects with evidence ratios of >19 are roughly similar to an alpha of 0.05 in null-hypothesis significance testing (NHST; Makowski et al., 2019; Milne and Herff, 2020).

We expect that high musical perception abilities transfer to stronger phonological processing which subsequently translates to higher performance in the CSWL task (as evidenced by higher accuracy), compared to those with less musical perception abilities. With regards to the three tested pair types, we expect them to follow the same pattern as in previous CSWL studies, namely, a higher performance for nonMPs and consMPs and lower performance for vowelMPs (Escudero et al., 2016a). Additionally, we were interested in the differences between the moderations of MPT and MDT per pair type. As the MPT tests for perception of fine-pitch changes, one might expect participants with higher MPT scores to learn vowel contrasts more easily due to the acoustic similarities between musical pitch and vowels. As MDT measures auditory short-term memory, we expect high MDT scores to positively correlate with accuracy in general.

## Results

**Figure 2:**
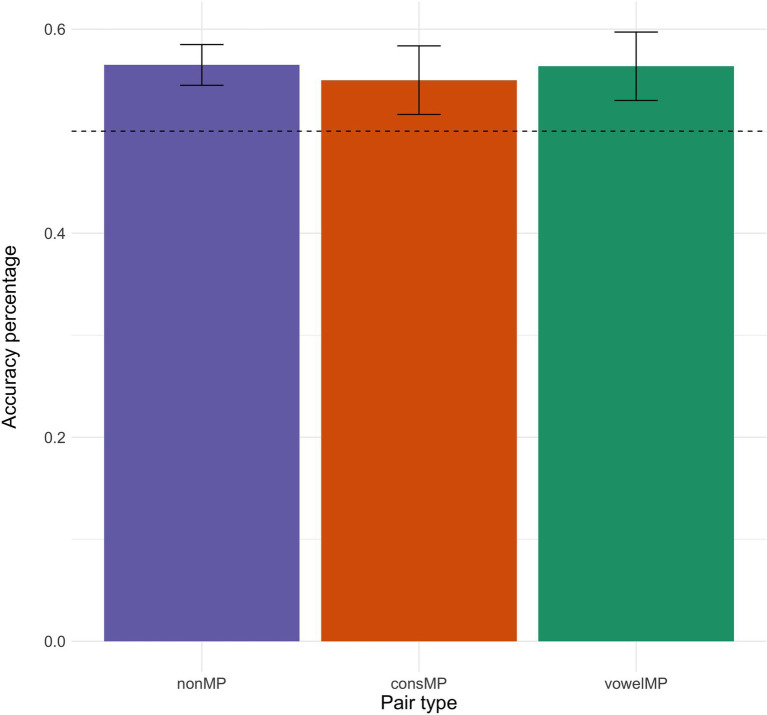
Mean accuracy (in percentage) per pair type. Error bars represent the standard error over the mean accuracy responses per pair type. The dotted line represents accuracy by chance.

Figure 2 shows the overall percentage of accurate responses per pair type. Performance across pair types appears to be very similar and participants were able to learn all pair types during the task, as evidence by performance being significantly above chance (see Figure 2). Accuracy for these learners is similar, albeit a little lower, to that found in a previous study (between 0.60 and 0.70 for all pair types) using the exact same design and online testing methodology (Escudero et al., 2021).

**Figure 3:**
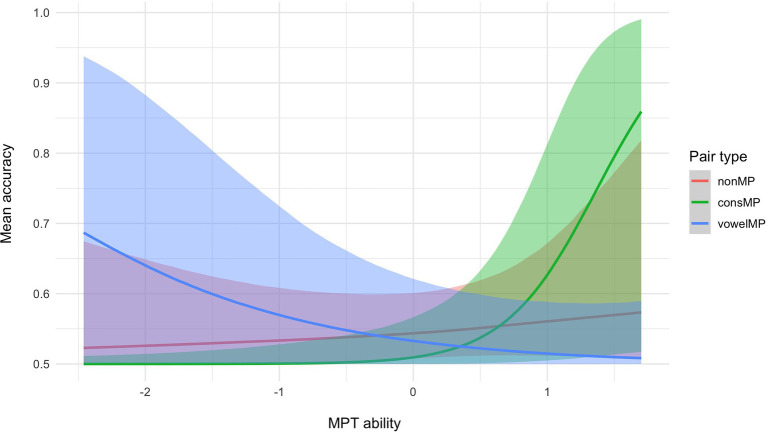
Conditional effects of MPT ability and pair type on mean accuracy with 95% credibility intervals.

**Figure 4:**
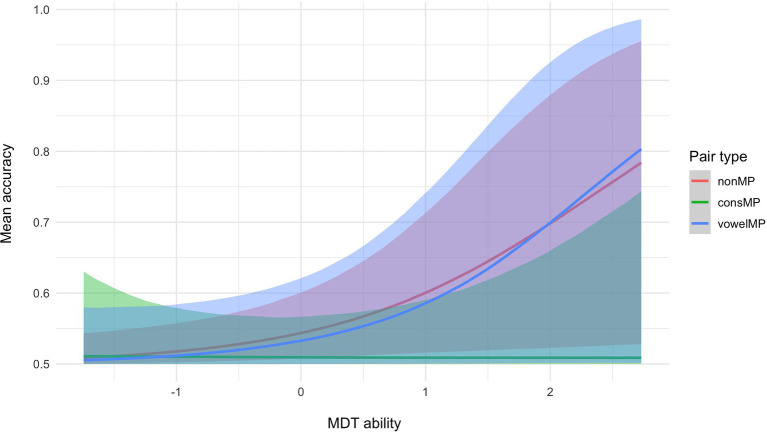
Conditional effects of MDT ability and pair type on mean accuracy with 95% credibility intervals.

Hypothesis tests run on the results from the multilevel Bayesian model show strong evidence that for participants with average MDT and MPT, accuracy for consMPs is lower than for nonMPs (see Table 1, hypothesis 1). We did not find sufficient evidence to support a difference between the other pair types (hypotheses 2 and 3). We then tested whether performance per pair type is moderated by MPT and MDT ability. As shown in Figure 3, mean accuracy for nonMPs does not appear to be moderated by MPT ability, whereas for consMPs, higher MPT ability leads to higher accuracy, which was not expected. Also unexpectedly, the opposite occurs for vowelMPs, where higher MPT ability negatively impacts performance. As per our predictions, for MDT ability (see Figure 4), we see that higher scores generally lead to improved accuracy, especially for nonMPs and vowelMPs. However, important to note is that, as visualized by the colored ribbons in Figures 3, 4, the slopes’ credibility intervals are highly overlapping, which indicates that the evidence for these differences might not be decisive. Therefore, we conducted hypothesis testing to confirm this (see hypotheses 4–6 for MPT ability and 10–12 for MDT ability in Table 1). As can be seen in Table 1, MDT ability influences accuracy in the expected direction (i.e., higher MDT leads to higher accuracy) for all pair types, but unexpectedly, MPT has a negative effect on accuracy for vowelMPs.

**Table 1:** Hypothesis testing—accuracy model.

Hypothesis tests	Estimate	Est. Error	[90% CI]	Evid. Ratio	Post. Prob
For average MDT and MPT ability:					
1. nonMP–consMP > 0	−1.83	1.67	[−4.88, 0.25]	11.11	0.92
2. nonMP–vowelMP > 0	−0.63	1.80	[−3.90, 1.21]	0.64	0.39
3. vowelMP–consMP > 0	1.20	2.47	[−2.63, 4.87]	3.27	3.27
*MPT ability > 0* in the following conditions and contrasts:					
4. nonMP	0.41	0.70	[−0.50, 1.76]	2.47	0.71
5. consMP	2.97	1.48	[0.80, 5.55]	91.78	0.99
6. vowelMP	−0.88	0.85	[−2.08, 0.17]	12.10	0.92
7. consMP–nonMP	2.55	1.53	[0.28, 5.20]	30.61	0.97
8. nonMP–vowelMP	1.30	1.05	[−0.07, 3.04]	16.37	0.94
9. consMP–vowelMP	3.85	1.69	[1.38, 6.68]	92.75	0.99
*MDT ability > 0* in the following conditions and contrasts:					
10. nonMP	0.95	0.42	[0.28, 1.63]	78.30	0.99
11. consMP	−0.08	0.84	[−1.45, 1.11]	0.92	0.48
12. vowelMP	1.16	0.93	[−0.07, 2.65]	16.33	0.94
13. nonMP–consMP	1.04	0.89	[−0.24, 2.52]	10.06	0.91
14. vowelMP–nonMP	0.21	0.98	[−1.78, 1.14]	1.44	0.59
15. vowelMP–consMP	1.24	1.23	[−0.54, 3.29]	7.63	0.88

*Estimate = mean of the effect’s posterior distribution. Estimate error = standard deviation of the posterior distribution. 90% CI = 90% credibility intervals. Evidence ratio = the posterior probability under the hypothesis against its alternative*.

Regarding the extent to which the effect of MPT and MDT differs by pair type, unexpectedly, we find very strong evidence that MPT ability has a stronger impact on accuracy for consMPs than for nonMPs and vowelMPs (see Table 1; hypotheses 7 and 9) and strong evidence for nonMPs compared to vowelMPs (see Table 1; hypothesis 8). Thus, not only does MPT negatively influence the learning of vowelMPs as shown in hypothesis 4, but it also impacts the learning of vowelMPs less strongly than the learning of nonMPs and consMPs. Our finding of strong evidence suggesting that MDT ability has a stronger impact on accuracy for nonMPs and vowelMPs compared to consMPs (see Table 1; hypotheses 13 and 15) was also unexpected, as we thought MDT would influence the learning of all pair types equally.

## Discussion

In this study, we tested whether music perception abilities impact the learning of novel word pairs in a CSWL paradigm that provides no explicit instruction during the learning phase. Overall, we found that participants were able to learn all novel word-object pairings regardless of the phonological overlap between the novel words, mostly replicating (albeit a little lower) previous reported results using the same online protocol (Escudero et al., 2021). That is, overall accuracy was comparable for novel words that had large phonological differences, forming non-minimal pairs (nonMPs), and for words that differed in a single consonant (consMPs) or a single vowel (vowelMPs). Regarding the relation between accuracy and music perception abilities, participants with average MPT and MDT had similar word learning scores across pair types, with performance for consMP probably being slightly lower than for the other pair types. Crucially, we found unexpected results for how MPT and MDT influenced word learning performance in nonMPs versus consonant and vowelMPs, which we discuss below.

As mentioned above, although we expected higher MPT participants to learn vowel contrasts more easily due to the acoustic similarities between pitched musical sounds and vowels (consonants do not have a clear pitch), we found the opposite effect. It appears that stimuli containing variable pitch information (such as vowels) pose extra difficulty for listeners who are more attuned to such information. A plausible explanation for these results is proposed by Ong et al. (2017b) who suggest that listeners’ experience is important for their ability to learn new acoustic cues, whether this experience is linguistic (through a native language that distinguishes lexical tone contrasts, such as Cantonese, Mandarin, or Thai) or musical. In a *distributional learning* (a form of statistical learning) experiment of nonnative lexical tones, they found that listeners without music or tonal language experience were able to discriminate lexical tones from ambiguous versions of the target tones after a short exposure (Ong et al., 2015). In a follow-up study, they found mixed results for *pitch experts*, who they define as listeners with extensive experience with pitch either through a tonal language or through musical training. Those with a tonal language background were able to learn non-native lexical tones distributionally but those with a musical background were not. This was unexpected as musical training has been found to have a positive effect on statistical learning (e.g., François et al., 2013; Chobert et al., 2014), and musicians were expected to perform better due to an improved ability to extract regularities from the input. These results led Ong and colleagues to conclude that domain-specific experience with pitch influences the ability to learn non-native lexical tones distributionally (Ong et al., 2017b), indicating no cross-domain transfer of music and linguistic abilities in distributional learning.

Ong and colleagues discussed their results in relation to the Second Language Perception (L2LP) model (Escudero, 2005; van Leussen and Escudero, 2015; Elvin and Escudero, 2019; Elvin et al., 2020, 2021; Yazawa et al., 2020), suggesting that the tonal language speakers only had to shift their category boundaries to the novel tonal categories, whereas the musicians had to create new categories, which is more difficult (Ong et al., 2017b). Another possible explanation is that musicians did not consider the stimuli as speech tones and thus may have processed them as musical stimuli resulting in them not learning the tonal categories (Ong et al., 2017b), but this argument assumes that musical pitch cannot be learned distributionally. In a different study, Ong et al., (2017a) tested distributional learning of musical pitch with nonmusicians and showed that they were able to acquire pitch from a novel musical system in this manner. This may be different for musicians, who were found to outperform nonmusicians in the discrimination and identification of Cantonese lexical tones (Ong et al., 2020).

From studies on distributional learning of pitch and lexical tones, it can be concluded that cross-domain transfer between speech and music largely depends on the listener’s musical or linguistic experience (Ong et al., 2015, 2016, 2017a,b, 2020). Nonmusicians without tonal language experience can learn novel pitch contrasts in both the speech and the music domain, but the situation is more complex for pitch experts, suggesting that those with extensive music experience may struggle more than those with tonal experience. However, an important difference between Ong et al.’s studies and the current study is that they tested listeners at both ends of the experience spectrum, while we tested listeners ranging from the lower to middle end of the music experience spectrum based on their music perception skills. By using music perception tasks, we were able to classify participants using a continuous predictor rather than splitting them into groups, which allowed us to uncover more detailed information about what happens with speech learning as music perception skills increase. A further difference is in the stimuli used, as the lexical and musical tones used in Ong et al. (2015, 2016, 2017a,b, 2020) contained many variable pitches along a continuum, while our stimuli had limited and uncontrolled pitch variation. Specifically, we focused on word learning of naturally produced novel words, where pitch variability was not consistent among the different words and pair types. Thus, listeners in the present study may have used other acoustic cues that are not pitch-related to discriminate and learn the novel words.

Given that listeners with strong pitch perception abilities are more likely to use pitch as a cue to discriminate between stimuli (Perfors and Ong, 2012; Ong et al., 2017b, 2020), our vowelMP stimuli may have been particularly challenging for them due to the use of infant-directed speech (IDS). IDS is the speech style or register typically used by mothers and caregivers when speaking to babies and is characterized by the use of larger pitch variations. Many studies have shown that IDS can facilitate word learning in infants (Ma et al., 2011; Graf Estes and Hurley, 2013) and adults (Golinkoff and Alioto, 1995) due to higher salience leading to enhanced attentional processing (Golinkoff and Alioto, 1995; Kuhl et al., 1997; Houston-Price and Law, 2013; Ellis, 2016). Despite it facilitating infant and adult speech learning, IDS may have a negative effect for those with strong musical perception abilities as they might think they are hearing different words due to varying pitch contours when only one word is presented. Unexpectedly, MPT ability affected learning of cMPS and nonMPs more than vMPs. As vMPs naturally contain more pitch variation, those were expected to be the most difficult to learn, hence the influence of IDS is likely stronger on cMPS and nonMPs than on vMPs. A similar result of hearing multiple words instead of one due to the use of IDS has been found in a prior CSWL study (Escudero et al., under review), where the target population consisted of native Mandarin speakers who were L2 English learners. Specifically, word pairs containing non-native vowel contrasts with IDS pitch fluctuations were difficult to learn for L1 Mandarin L2 English learners.

Thus, in populations where pitch variations indicate different lexical meanings, such as native speakers of Mandarin (Han, 2018), IDS can be problematic and impair word learning as participants might perceive multiple categories where only one is presented (Escudero and Boersma, 2002; Elvin et al., 2014; van Leussen and Escudero, 2015). The impact of a learner’s native language on novel language learning has been explained by L2 speech theories (e.g., Flege, 1995; Escudero, 2005; Best and Tyler, 2007; van Leussen and Escudero, 2015). In particular, the L2LP model (Escudero, 2005; van Leussen and Escudero, 2015; Elvin and Escudero, 2019; Elvin et al., 2020, 2021; Yazawa et al., 2020) proposes three learning problems when L1 and L2 categories differ in number or in phonetic realization. This model is the only one that handles lexical development and word learning with consideration of hearing more differences than produced in the target language as a learning problem (van Leussen and Escudero, 2015; Escudero and Hayes-Harb, 2021). Specifically, listeners can categorize binary L2 contrasts into more than two L1 categories, which is referred to as Multiple Category Assimilation (MCA, L2LP; Escudero and Boersma, 2002) and can lead to a *subset problem* (Escudero and Boersma, 2002; Escudero, 2005; Elvin and Escudero, 2014, 2019). A subset problem occurs when an L2 category does not exist in a listener’s L1 but is acoustically similar to two or more separate L1 categories and thus is perceived as more than one L1 sound, with no overt information from the target language that will allow the learner to stop hearing the extra category or stop activating *irrelevant* or *spurious* lexical items (Escudero and Boersma, 2002; Escudero, 2005; Elvin and Escudero, 2014, 2019).

With regard to our CSWL task, we expect that using *adult-directed speech* (ADS) without these additional pitch fluctuations would improve learning for the nonMPs and consMPs for tonal language speakers, but not for vowelMPs. When using IDS, nonmusicians and non-tonal speakers show a pattern where performance is lowest for pair types with the highest pitch variability (i.e., vowelMPs). The use of IDS, which adds even more pitch variability than naturally present in the vowelMPs, seems to pose problems for learners who are not music experts but have some music perception skills. For tonal language speakers, the use of IDS poses problems in general as they consistently use pitch information to discriminate between all pair types. If pitch variability is the main predictor for performance in this CSWL task, then music experts (i.e., musicians) should struggle more with the vowelMPs than the nonmusicians tested here but should perform better for the nonMPs and consMPs than the tonal language speakers discussed earlier in Escudero et al. (under review).

Regarding the results for MDT, although not decisive, the evidence suggests that MDT ability more strongly influences accuracy for nonMPs and vowelMPs compared to consMPs. The MDT ability test focuses heavily on auditory short-term memory (Dowling, 1978; Harrison et al., 2017; Harrison and Müllensiefen, 2018). It has been suggested that auditory short-term memory for consonants is distinct from that for vowels (Pisoni, 1975), as explained by the cue-duration hypothesis (Pisoni, 1973), which suggests that the acoustic features needed to discriminate between two different consonants are shorter and thus less well represented in auditory short-term memory than those of vowels (Chen et al., 2020). As well, seminal studies on speech sounds have suggested that consonants may be stored differently in short-term memory compared to vowels (Crowder, 1971, 1973a,b), with the idea that vowels are processed at an earlier stage compared to consonants (Crowder and Morton, 1969). It is possible that a different type of auditory memory is activated for nonMPs, which does not rely as strongly on the discrimination of the acoustic features of the stimuli than what is needed to distinguish between phonologically overlapping stimuli. As similarly suggested in Escudero et al. (2021), this could be tested using time-sensitive neurophysiological methods, such as electroencephalography (EEG).

Some limitations of this study must be noted. Even though we tested for perceptual skills, it is possible that accuracy also depends on other skills, such as how well a listener is able to do crossmodal associations. Likewise, it is possible that general cognitive abilities may impact the learning of novel words in an ambiguous word learning paradigm. As we find some differences between accuracy for the different pair types in the current study and prior CSWL studies using the same paradigm (Escudero et al., 2016; Mulak et al., 2019), it might seem that individual differences, such as the ability to do crossmodal associations or general cognitive abilities, may be the cause of these differences. However, there are other possible sources between the current study and prior CSWL results that might have led to the differences between studies, such as the number of trials and the number of responses used in the learning and test phases. We are currently replicating learning and testing phases from those previous studies using online testing to see if the number of trials is the source of the difference. If this is not the case, future studies can then look further into other possible sources, such as general cognitive abilities. Regarding the use of IDS, it is an empirical question whether adults in general will perform better with stimuli characterized by shorter durations, and non-enhanced differences between vowels and neutral prosodic contours (such as ADS). On the contrary, we found that enhanced vowel differences that are similar to those typical of IDS facilitate phonetic discrimination for adults listeners (Escudero et al., 2011; Escudero and Williams, 2014). Additionally, there is a possibility that the degree of novelty of the auditory and visual stimuli impacts accuracy responses. Even though language background did not have an influence on accuracy, future studies could consider implementing measuring participants’ familiarity with the stimuli. Another possible limitation is that we did not collect information regarding participants’ headphones. However, we did check whether participants were able to hear the stimuli and were wearing headphones, as part of our pre-registered protocol.

Overall, the results show that the tested music perception abilities impact the learning of words that differ in a single consonant or vowel differently and in complex ways. Pitch perception is an important factor for novel word learning, to the extent that those with stronger pitch perception skills are better at distinguishing consonant contrasts, and apparently *too* good at distinguishing vowel contrasts. Using stimuli produced in adult-directed-speech, our follow-up research will establish whether the negative correlation between pitch perception and accuracy in words distinguished by a single vowel is due to our use of IDS and its concomitant large pitch variations. We also find that consonants and vowels are learned differently for those with melodic discrimination skills, reflected in improved auditory short-term memory. In contrast to MPT, an increase in MDT leads to better learning of words distinguished by a single vowel than those distinguished by a single consonant, which may be connected to better auditory short-term memory for vowels. The contrasting results for the two tested music perception skills may reflect different stages of processing. Our results have one clear implication for theories of cross-domain transfer between music and language: considering populations along the entire spectrum of musicality and linguistic pitch experiences is the only way to uncover exactly where and when problems with word learning occur.

## Conclusion

We tested whether specific music perception abilities impact learning of minimal pair types in adults that have not been selected for their musical abilities. Using a CSWL paradigm, we have shown that pitch perception and auditory working memory affect the learning of vowel and consonant minimal word pairs, but vowels and consonants are impacted differently. We suggest this may be due to the pitch fluctuations of the specific characteristic of stimuli, namely, words produced in infant-directed speech (IDS). Similar to the patterns observed in native speakers of tonal languages, this type of speech register may lead to the listeners’ perception of more distinctions than intended. In future studies, we aim to test the role of IDS compared to adult-directed speech, how specific levels of training in music impact performance in CSWL, and the differential storage of vowels versus consonants.

## Data Availability Statement

The raw data supporting the conclusions of this article will be made available by the authors, without undue reservation.

## Ethics Statement

The studies involving human participants were reviewed and approved by the Western Sydney University Human Research Ethics Committee (H11022). The participants provided their written informed consent to participate in this study.

## Author Contributions

ES and PE conceived the initial experiments. ES was responsible for overseeing data collection and wrote the initial draft. ES and AM analyzed the data. ES, AM, and PE wrote the paper. All authors contributed to the article and approved the submitted version.

## Funding

Data collection was funded by a Transdisciplinary & Innovation Grant from the Australian Research Centre for the Dynamics of Language (project number TIG1112020) awarded to ES. PE’s and ES’ work and the article publication fees were funded by an Australian Research Council Future Fellowship (FT160100514) awarded to PE. AM’s work was funded by an Australian Research Council Discovery Early Career Researcher Award (project number DE170100353).

## Conflict of Interest

The authors declare that the research was conducted in the absence of any commercial or financial relationships that could be construed as a potential conflict of interest.

## Publisher’s Note

All claims expressed in this article are solely those of the authors and do not necessarily represent those of their affiliated organizations, or those of the publisher, the editors and the reviewers. Any product that may be evaluated in this article, or claim that may be made by its manufacturer, is not guaranteed or endorsed by the publisher.
